# Antiviral Activity of Graphene–Silver Nanocomposites against Non-Enveloped and Enveloped Viruses

**DOI:** 10.3390/ijerph13040430

**Published:** 2016-04-19

**Authors:** Yi-Ning Chen, Yi-Huang Hsueh, Chien-Te Hsieh, Dong-Ying Tzou, Pai-Ling Chang

**Affiliations:** 1 Department of Bioscience Technology, Chung Yuan Christian University, 200 Chung Pei Road, Chung Li District, Taoyuan City 32023, Taiwan; 2 Graduate School of Biotechnology and Bioengineering, Yuan Ze University, 135 Yuan-Tung Road, Chung Li District, Taoyuan City 32003, Taiwan; yihhsueh@saturn.yzu.edu.tw; 3 Department of Chemical Engineering and Materials Science, Yuan Ze University, 135 Yuan-Tung Road, Chung Li District, Taoyuan City 32003, Taiwan; cthsieh@saturn.yzu.edu.tw (C.-T.H.); dong1015211@gmail.com (D.-Y.T.); 4 Division of General Pathology, Taoyuan General Hospital, Ministry of Health and Welfare, 1492 Zhongshan Road, Taoyuan City 33004, Taiwan; patty37021@mail.tygh.gov.tw

**Keywords:** graphene oxide, silver nanoparticle, feline coronavirus, infectious bursal disease virus, enveloped virus, non-enveloped virus

## Abstract

The discovery of novel antiviral materials is important because many infectious diseases are caused by viruses. Silver nanoparticles have demonstrated strong antiviral activity, and graphene is a potential antimicrobial material due to its large surface area, high carrier mobility, and biocompatibility. No studies on the antiviral activity of nanomaterials on non-enveloped viruses have been reported. To investigate the antiviral activity of graphene oxide (GO) sheets and GO sheets with silver particles (GO-Ag) against enveloped and non-enveloped viruses, feline coronavirus (FCoV) with an envelope and infectious bursal disease virus (IBDV) without an envelope were chosen. The morphology and sizes of GO and GO-Ag were characterized by transmission, scanning electron microscopy, and X-ray diffraction. A virus inhibition assay was used to identify the antiviral activity of GO and GO-Ag. Go-Ag inhibited 25% of infection by FCoV and 23% by IBDV, whereas GO only inhibited 16% of infection by FCoV but showed no antiviral activity against the infection by IBDV. Further application of GO and GO-Ag can be considered for personal protection equipment to decrease the transmission of viruses.

## 1. Introduction

Various emerging infectious diseases caused by viruses, including severe acute respiratory syndrome coronavirus (SARS-CoV), Ebola virus, norovirus, and dengue virus have prompted the discovery and development of antimicrobial reagents and personal protection equipment (PPE) to guard against infectious agents. Silver nanoparticles (Ag NPs) have been proven to be the most effective antimicrobial agents against bacteria and viruses because of their high surface-area-to-volume ratio and unique chemical and physical properties, even though they have shown cytotoxicity at high concentrations [1,2,3]. Limited studies have found that Ag NPs show antiviral activity against human immunodeficiency virus [1,4,5,6], hepatitis B virus [7], herpes simplex virus type 1 [8], respiratory syncytial virus [9], monkey poxvirus [10], Tacaribe virus [11], and H1N1 influenza A virus [12,13,14]. All the RNA and DNA viruses mentioned previously contain lipid envelopes. No antiviral effects of Ag NPs on non-enveloped viruses have been investigated.

The major antiviral mechanism of Ag NPs has not been investigated extensively, but the most frequently observed method by which Ag NPs may inhibit viruses is the physical binding between the virus and Ag NPs to block the entry of the viruses into cells. Ag NPs could inhibit the *in vitro* production of HBV RNA and extracellular virions by interacting with the HBV viral particles, as revealed by transmission electronic microscopy (TEM) [7]. At an early stage of viral replication, Ag NPs are observed by TEM to bind to glycoprotein 120 and thus prevent the binding and fusion of HIV-1 and CD4 cells. In addition, Ag NPs can inhibit the post-entry stages of the HIV-1 life cycle [5]. Because Ag NPs have a natural tendency to bind the disulfide bonds in each monomer of hemagglutinin protein on the surface of an influenza virus and block its host receptor binding sites, Ag NPs can inhibit the absorption of influenza viruses to Madin–Darby canine kidney (MDCK) cells and chicken red blood cells [12]. Numerous factors can determine the antiviral efficacy of Ag NPs, including size, shape, and capping agents. Previous studies have found that the most effective size of Ag NPs is <10 nm [14] and that a spherical shape is superior to a tubular shape or aggregation. Furthermore, previous studies have revealed that capping agents limiting the release of free Ag NPs would exhibit lower viral inhibition [5]. Therefore, novel material for the capping and support agents of Ag NPs is required for a superior application of Ag NPs.

Graphene, a single atomic plane of graphine with 2-dimensional extension, is promising as a next-generation nanomaterial due to its unique high carrier mobility, effective optical transparency, large surface area, and biocompatibility [15]. GO is employed in the production of graphene family nanomaterials for various applications. The antibacterial activity of well-dispersed GO sheets has been evaluated [16,17,18], and a few studies have reported that graphene-based material can inhibit the entry and replication of enveloped DNA virus (herpesvirus) and RNA virus (coronavirus) in their target cells [19,20]. The pertinent findings of previous studies on the nanocomposites formed by Ag NPs and GO sheets against bacteria can be applied to investigations of the antiviral activity of nanocomposites composed of GO sheets and Ag NPs (GO-Ag). The GO sheets can serve as a supporting and stabilizing agent in preventing the agglomeration of the Ag NPs and consequently in preventing a reduction of the antibacterial activity. The Ag NPs supported on GO sheets showed a spherical-like morphology and an average size of 7.5 nm [16], which is suitable for antiviral activity [14]. The most significant advantage of GO-Ag nanocomposites over free Ag NPs is that the immobilization of Ag NPs on GO sheets prevents the movement of the nanoparticles, thus increasing the material’s biocompatibility and reducing the toxicological effects and the environmental impact associated with metallic nanoparticles. In addition, the GO-Ag matrix is highly dispersible in water, contains a large specific surface area, shows excellent bactericidal activity at extremely low concentrations, and exhibits no corrosive characteristics.

In the present study, feline coronavirus (FCoV) and infectious bursal disease virus (IBDV) were respectively chosen as enveloped and non-enveloped viruses to test the antiviral activity of nanocomposites composed of GO-Ag. Feline CoV is a positive-sense, single-stranded RNA virus with a lipid envelope and belongs to the family *Coronaviridae*. It usually causes feline infectious peritonitis—a progressive and fatal disease in cats—and is transmitted through direct contact with secretions [21]. IBDV is a double-stranded RNA virus without an envelope and belongs to the genus *Avibirnavirus* of the family *Birnaviridae*. It mainly infects chickens, leading to immunosuppression and significant economic loss [22]. If the GO-Ag shows promising antiviral activity, then we hope to apply this novel material to manufacturing personal protection equipment (PPE) to protect against viral infections.

## 2. Materials and Methods

### 2.1. Preparation of Graphene Oxide (GO) Sheets

Graphene oxide (GO) sheets were prepared according to Hummers’ method [23]. First, graphite powder and sodium nitrate were mixed with sulfuric acid in an ice bath with agitation for 4 h. Next, potassium manganate was added under stirring and the mixture was kept at 35 °C for 2 h. Then, the mixture was diluted with distilled water and kept at 95 °C for another 2 h before the further dilution with warm water. After the mixture turned yellow with stirring, the mixture was centrifuged to obtain GO powders. The GO powders were re-dispersed in water, and the solution was sonicated for 2 h to facilitate the exfoliation of stacked GO into monolayer or multilayered GO sheets about several micrometers in lateral size. The GO was further reduced using beta-mercaptoethanol, and the black powders of the final product were recovered after centrifugation.

### 2.2. Preparation of Silver Nanoparticles Anchored Graphene Oxide (GO-Ag)

GO powders were dispersed in the silver-containing solution, which consisted of AgNO_3_ (0.5 M, 35 mL) and ethylene glycol (EG, 70 mL). After the solution was mixed, pulse microwave-assisted (MA) synthesis was processed by placing the solution in the microwave oven (Tatung Co., Taipei City, Taiwan, 900 W, 2.45 GHz) at 160 °C for 5 min to ensure the growth of silver seeds deposited on the GO surface. After GO-Ag solution was produced, it was dried in a vacuum oven at 60 °C overnight.

### 2.3. Characterization of GO and GO-Ag

GO and GO-Ag were prepared by grinding and drying in a vacuum oven at 80 °C for further characterization. The morphology of GO and GO-Ag and the distribution of Ag particles on GO sheets were observed by using high-resolution transmission (HR-TEM, JEOL, Tokyo, Japan, 40 kV, JEM-2100) and field-emission scanning electron microscopes (FE-SEM, JEOL, 15 kV, JSM-6701F). The sample was placed on 300-mesh copper grids for HR-TEM and embedded in a copper paste for FE-SEM. Crystalline of GO and chemical composition were carried out via X-ray diffraction (XRD, Shimadzu, Kyoto, Japan, XRD-6000, Cu-Kα radiation, λ = 0.154 nm) and X-ray photoelectron spectrometer (XPS, Thermo Scientific, Waltham, MA, USA, K-Alpha). The thickness and layer number of GO were scanned and analyzed with an atomic force microscope (AFM, Minus K Technology, Inglewood, CA, USA, P100). The sample was dissolved in ethanol, spread on silicon wafer, and dried in a vacuum oven at 80 °C for AFM. A thermogravimetric analyzer (TGA, PerkinElmer, Waltham, MA, USA, Pyris 1 TGA) was used to calculate the loading (wt %) of Ag nanoparticles on GO sheets. The TGA analysis was executed under an air atmosphere and GO-Ag was heated at the rate of 10 °C/min. The carbon and oxygen of the GO-Ag were released in the forms of CO_2_ and water after heating, and the remaining weight of Ag could be used to calculate the loading (weight %) of Ag particles in 1 g of GO sheet.

### 2.4. Cell Culture

To understand the effects of GO and GO-Ag on the enveloped and non-enveloped viruses, FCoV and IBDV are chosen. *Felis catus* whole fetus-4 (fcwf-4) cells for FCoV [21] and DF-1 cells for IBDV were maintained in Dulbecco’s Modified Eagle Medium (DMEM) with 10% fetal bovine serum (FBS), 100 IU/mL penicillin, and 100 IU/mL streptomycin solution in 5% CO_2_ at 37 °C. DF-1 cell is an immortalized cell line of chicken embryo fibroblasts (CEF), which can support the growth of several avian viruses including IBDV [22].

### 2.5. Viruses and Virus Titration

Feline CoV/NTU156 was propagated in fcwf-4 cells at a multiplicity of infection (MOI) of 10 [21] and IBDV was propagated in DF-1 cells at a MOI of 0.1 [22]. Cytopathic effects (CPE) caused by FCoV and IBDV were observed and recorded. After 96 h of incubation, FCoV and IBDV were harvested. The extracellular virus were collected after the centrifugation at 500× *g* for 5 min. Cell-associated FCoV and intracellular IBDV were released via three frozen-thawed cycles. The virus titer was determined with a tissue culture infectious dose (TCID) assay. The cells were inoculated to 96-well plates in the concentration of 4 × 10^4^ cells in 100 μL per well and reached confluent monolayer after 24 h of incubation. After discarding old growth media in wells, 50 μL of the 10-fold diluted virus solution was added into each well. Each dilution of virus had 8 wells for inoculation, and every TCID assay was performed in duplicate. After the absorption of the virus for 2 h at 37 °C, 50 μL of fresh media were added in each well and incubated for 96 h in 5% CO_2_ at 37 °C. The infectivity of viruses was determined by observing the formation of CPE and the staining of viable cells by 1% crystal violet. The cells stained with 1% crystal violet were not infected by viruses, and the infected cells were washed away after the staining. The infectivity of viruses was calculated as followed: infectivity % = (number of wells with virus-infected cells/number of wells with virus-inoculated cells) × 100%. By using the infectivity percentages, the virus dilution can infect 50% of cells. TCID_50_, was calculated as followed: ((infectivity % at dilution immediately above 50%) − 50%)/((infectivity % at dilution immediately above 50%) − (infectivity % at dilution immediately below 50%)) [24].

### 2.6. Virus Inhibition Assay

To determine the antiviral activity of GO and GO-Ag, serially diluted GO or GO-Ag solutions were incubated with serially diluted solutions of FCoV or IBDV at 37 °C for 1 h. Then, the mixture was centrifuged at 6000 rpm for 10 min to remove the composite particles. The supernatant (50 μL) will be transferred to 96-well plate with cells in each well and incubated at 37 °C and 5% CO_2_ for 1 h, followed by the addition of fresh media (50 μL) and the incubation. The infected cells will be observed every day for CPE by viral infection. After 96 h, the cells were fixed with methanol and stained with 5% of crystal violet solution in methanol. The antiviral activity of GO and GO-Ag was determined as the ratio of TCID_50_/mL from the group of GO or GO-Ag treatment to TCID_50_/mL from the group of virus-only suspension. The inhibition efficacy was calculated as followed: inhibition % = (log_10_ (TCID_50_/mL of virus-only group) − log_10_ (TCID_50_/mL of GO or GO-Ag treatment with virus group))/log_10_ (TCID_50_/mL of virus-only group) × 100%. The minimum inhibitory concentration is the lowest concentration of GO or OG-Ag can inhibit the infection of the virus completely after serial dilutions.

### 2.7. Determination of Cytotoxicity of GO or GO-Ag

Cytotoxicity of GO or GO-Ag to fcwf-4 cells was determined in terms of the concentration of GO or GO-Ag, causing 50% of cells to die (cytotoxicity concentration, CC_50_), by using MTS assay (CellTiter 96**^®^** AQueous One Solution Cell Proliferation Assay, Promega, Madison, WI, USA) for measuring the activity of cellular enzymes that reduce the tetrazolium dye to its insoluble formazan. The assays measured cellular metabolic activity via NAD(P)H-dependent cellular oxidoreductase enzymes and reflect the number of viable cells present. After the cells were incubated with the serially diluted GO or GO-Ag solutions for 1 h or 24 h at 37 °C, the enzymes were added into the treated cells to release the color from the cells. After 3 h of incubation, optical density can be measured at 500 nm using BioTek Synergy multi-detection microplate reader (BioTek, Winoosk, VT, USA), and CC_50_ can be calculated. All assays were performed in triplicate. Comparisons of results between GO and GO-Ag groups were analyzed using a *t*-test in the R program [25]. *p*-values of <0.05 were regarded as statistically significance.

## 3. Results

### 3.1. Characterization of GO and GO-Ag

**Figure 1:**
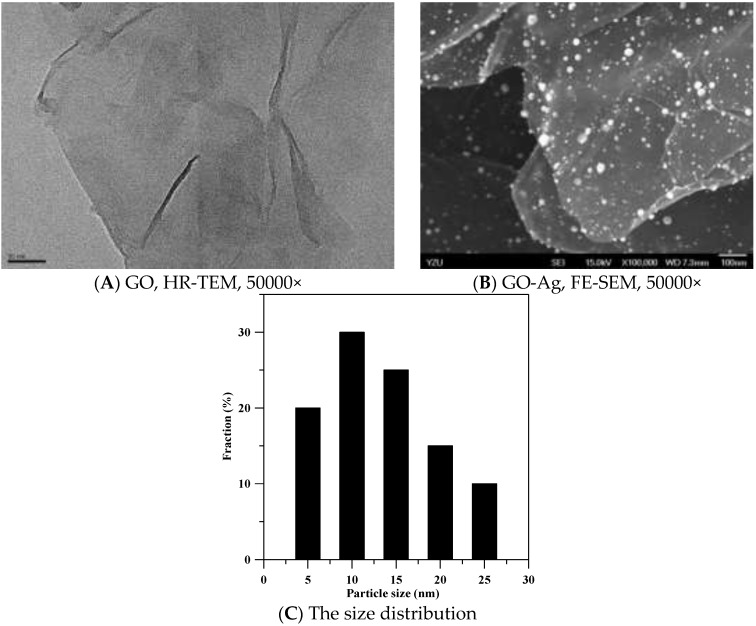
Morphology of graphene oxide sheets (GO) and silver nanoparticles on GO sheets (GO-Ag). (**A**) Image of GO sheets under high-resolution transmission electron microscopes (HR-TEM) at 50,000×; (**B**) image of GO-Ag under field-emission scanning electron microscopes (FE-SEM) at 50,000×; (**C**) the size distribution of Ag particles on GO sheet.

**Figure 2:**
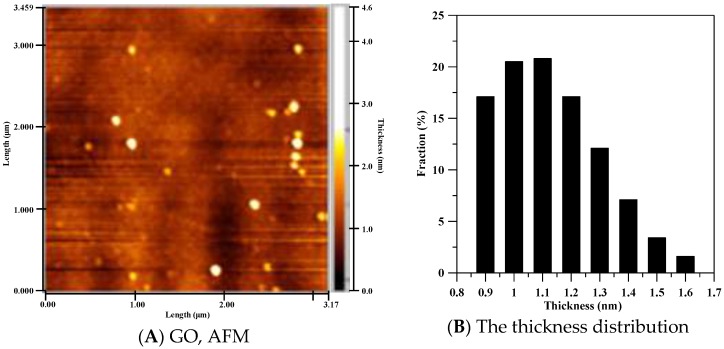
Thickness of graphene oxide (GO) sheets. (**A**) Image of GO sheets under atomic force microscope (AFM); (**B**) the thickness distribution of GO sheet.

The final product of GO and GO-Ag is black powder and needs to be re-suspended in PBS or cell growth media for tests. Shown in Figure 1, the HR-TEM image of GO revealed a thin transparent layer of GO sheet and a smooth surface with small wrinkles and folded edges (Figure 1A). The FE-SEM image of GO-Ag displays spherical-like particles decorating GO sheets, indicating that Ag particles were successfully attached and evenly dispersed on the GO sheets (Figure 1B). The size distribution of Ag particles was between 5 and 25 nm (Figure 1C). The loading of Ag particles calculated by TGA was 10% per gram of GO sheet. Based on the image of AFM (Figure 2A), the thickness distribution of GO was between 0.6 and 9 nm (Figure 2B). When the thickness of the GO monolayer is 0.355 nm, the GO sheets produced in our study had 2 to 5 layers.

**Figure 3:**
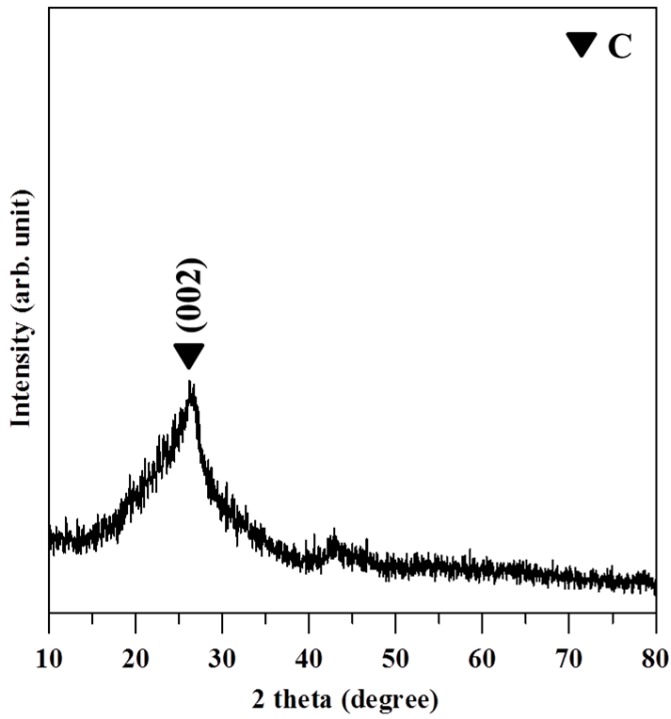
X-ray diffraction (XRD) pattern of reduced GO shows the strongest peak at 25.8° and the distance of d (002) is 0.348 nm.

**Figure 4:**
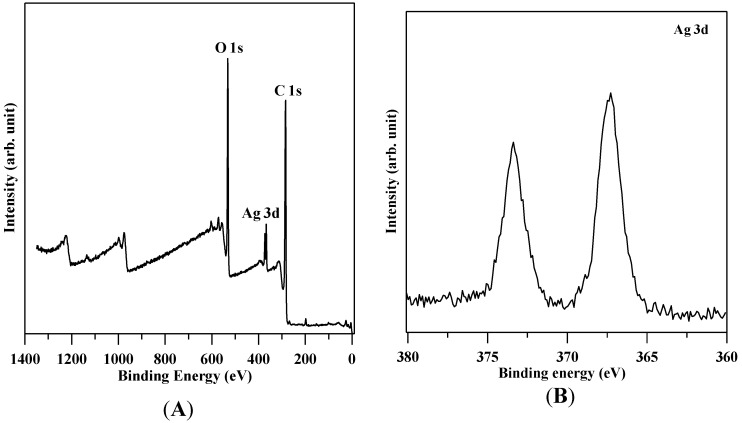
X-ray photoelectron spectrometer (XPS) patterns. (**A**) O/C ratio of reduced graphene oxide (GO) is 0.326; (**B**) Ag nanoparticles are deposited on GO.

The XRD pattern of GO shown in Figure 3 revealed a broad diffraction peak from 24° to 29°. The pattern suggests that the GO sheets were reduced, and some oxygen-containing functional groups were removed during the process of reduction. The broad diffraction peak indicates that the sample was poorly ordered along the stacking direction and comprised largely free nano-sheets. The XPS patterns in Figure 4 showed that the O/C ratio of GO is 0.326 and also confirmed that Ag nanoparticles are deposited on GO.

### 3.2. Cytotoxicity of GO and GO-Ag

**Figure 5:**
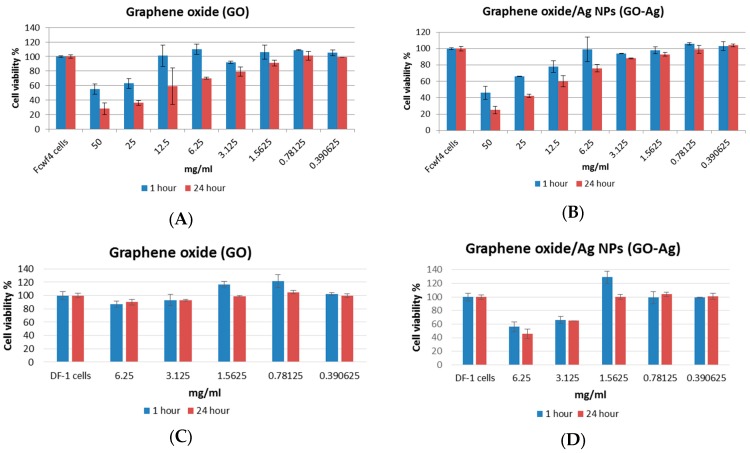
Viability of cells in the presence of graphene oxide (GO) and GO with silver nanoparticles (GO-Ag). (**A**) GO in fcwf-4 cells; (**B**) GO-Ag in fcwf-4 cells; (**C**) GO in DF-1 cells; (**D**) GO-Ag in DF-1 cells.

Viability of fcwf-4 cells was determined after 1 h and 24 h of incubation with two-fold serially diluted GO or GO-Ag from 50 mg/mL (Figure 5A,B). Besides the highest concentration of 50 mg/mL, GO-Ag had less cytotoxicity than GO in fcwf-4 cells without any statistical significance (*p* > 0.05). When the concentration of GO or GO-Ag was less than 1.5625 mg/mL, the cell viability was more than 90% after 1 h and 24 h of incubation. The CC_50_ of GO and GO-Ag was 17.4 mg/mL and 19.7 mg/mL in fcwf-4 cells, respectively. Viability of DF-1 cells was determined after 1 h and 24 h of incubation with two-fold serially diluted GO or GO-Ag from 6.25 mg/mL (Figure 5C,D). At the concentration of 6.25 and 3.125 mg/mL, GO-Ag showed significantly more cytotoxicity than GO (*p* < 0.05) after 1 h and 24 h of incubation. At a concentration of 1.625 mg/mL or lower, GO and GO-Ag did not have any toxic effect on DF-1 cells. The CC_50_ of GO and GO-Ag was over 6.25 mg/mL and 5.6 mg/mL in DF-1 cells, respectively.

### 3.3. Cytopathic Effect (CPE) of the Viruses

**Figure 6:**
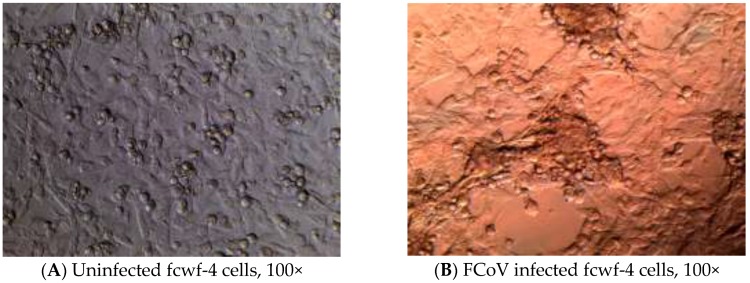

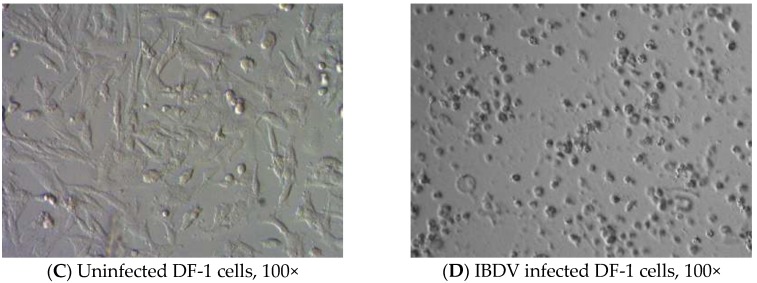
Cytopathic effects (CPE) of feline coronavirus (FCoV) with a lipid envelope and infectious bursa virus (IBDV) without a lipid envelope. (**A**) *Felis catus* whole fetus-4 (fcwf-4) cells without virus, monolayer at 24 h, 100×; (**B**) Fcwf-4 cells infected with FCoV/NTU156 become multinucleated giant cells (arrows) at 24 h post-inoculation (hpi), 100×; (**C**) DF-1 cells without virus, monolayer at 24 h, 100×. (**D**) DF-1 cells infected with IBDV have granulation around nuclei, cell rounding, and detachment at 72 h, 100×.

Multinucleated giant cells due to the fusion between cells are the typical CPE of coronavirus infection, including FCoV (Figure 6B). Syncytial cells can be observed as early as 18 h post-inoculation (hpi), followed by cell rounding and eventual detachment. Infection of IBDV in DF-1 cells produced typical CPE, characterized by a granulation around nuclei, cell rounding, followed by fragmentation of cells into small particles, and finally detachment until the entire monolayer was destructed (Figure 6D).

### 3.4. Virus Inhibition of GO and GO-Ag

Both GO and GO-Ag showed concentration-dependent activity to inhibit the infection of FCoV in fcwf-4 cells (Table 1). The higher inhibition percentage on the infection of FCoV can be observed with the lower concentration of FCoV and the higher concentration of GO and GO-Ag. In addition, GO-Ag can inhibit the infection of FCoV better than GO. With 0.1 mg/mL of GO-Ag, 24.8% of infection by 4.7 × 10^4^ TCID_50_/mL of FCoV was inhibited. From the data shown in Table 1 (Exp 3) and Table 2, no inhibition of vira;;;;l infection was observed after the incubation of IBDV nor any concentration of GO in DF-1 cells. Only GO-Ag had a concentration-dependent ability to inhibit the infection of IBDV in DF-1 cells. The higher concentration of GO-Ag with the lower concentration of IBDV infected fewer DF-1 cells. With 1 mg/mL of Go-Ag, 22.7% of infection by 9 × 10^5^ TCID_50_/mL of IBDV in DF-1 cells was inhibited (Table 1, Exp 3). The minimum inhibitory concentration of GO-Ag was 0.125 mg/mL against the infection of 9 × 10^2^ TCID_50_/mL IBDV and was higher than 1 mg/mL against the infection of 9 × 10^3^ TCID_50_/mL IBDV in DF-1 cells (Table 2).

## 4. Discussion

This is the first report investigating the antiviral activity of GO and GO-Ag against enveloped and non-enveloped viruses. Ag NPs have shown antiviral activity against enveloped viruses in previous studies, but no studies have discussed the antiviral activity of GO and GO-Ag. The antiviral mechanisms of Ag NPs are mainly based on the blocking of viral entry and the interference with viral membrane fusion [12,14]. The antiviral activity of GO-Ag observed in the current study should be contributed to by Ag particles on GO sheets. The sizes of Ag particles on GO sheets shown by FE-SEM images ranged from 5 to 25 nm, and about 50% of Ag particles were 10 nm or fewer than 10 nm. Less inhibition ability (25%) of GO-Ag (1 mg/mL) against coronavirus (47,000 TCID_50_/mL) was observed, compared to 97% of inhibition against influenza virus (100 TCID_50_/mL) by Ag NPs (0.05 mg/mL) [14]. However, the virus inhibition ability of Ag NPs in Xiang’s study [14] is not much better than that of GO-Ag in this study if we calculate the differences of virus and compound concentrations. Since GO sheets also showed antiviral ability against the coronavirus in this study, the synergistic effect from GO sheets and Ag particles on the inhibition against the infection of an enveloped virus should be considered.

No studies have been done on the antiviral activity of GO and GO-Ag but strong antibacterial activity has been observed in GO and GO-Ag. Exposure to GO and reduced GO can induce significant production of reactive oxygen species in *Pseudomonas* and lead to the fragmentation of bacterial DNA and death [17]. GO-Ag can disrupt the cell wall of Gram-positive bacteria and inhibit cell division of Gram-negative bacteria [26]. From de Faria’s study, GO sheets showed no antibacterial activity, while GO-Ag had a 100% inhibition rate of the adhered cells, thus preventing the process of biofilm formation [16]. Because of the differences of antiviral activity against enveloped and non-enveloped viruses by GO sheets, it is reasonable to assume that there is a physical or chemical interaction between GO sheets and the envelope of coronavirus leading to decreased infectivity. Without an envelope, the antiviral activity would solely rely on Ag particles, but GO sheets can boost the antiviral effect of Ag particles by supporting the even dispersion of Ag particles and the formation of spherical particles without aggregations. Additionally, the immobilization of Ag NPs on GO sheets has reduced cytotoxicity, usually caused by free Ag NPs.

Silver particles anchored on graphene oxide sheets (GO-Ag) can inhibit the infectivity of enveloped and non-enveloped viruses with very low to none cytotoxicity to cells susceptible to viruses. For non-enveloped viruses, graphene oxide (GO) sheets act as supporting materials for antiviral Ag particles without any antiviral ability. For the enveloped virus, GO sheets not only can be used to assist the even dispersal of Ag particles but also have ability to inhibit the infection of viruses. GO sheets also show very low cytotoxicity to cells. In conclusion, this study discovered that GO and GO-Ag are novel materials for the further development of protective reagents and equipment against the transmission of infectious viruses.

Because both enveloped and non-enveloped viruses can cause severe infectious diseases, we choose one to test GO and GO-Ag. In consideration of strict regulation of BSL-3 pathogens and the dangers of handling, we chose feline coronavirus (FCoV) for the enveloped virus and infectious bursal disease virus (IBDV) for the non-enveloped virus, because no zoonotic transmission by them has ever been reported. The protective effect of masks such as N95 and three-layer surgical masks is only maintained when the surface layer of the mask is hydrophobic and dry. The protective effect of the N95 facemask was reduced significantly when the mask was moistened by accidental splashes of blood or body fluids from patients, or by sweat and respiratory droplets from the wearers. If the mask surface is contaminated with infectious agents, microorganisms may be able to penetrate the protective layers along with the droplets [27]. Novel GO-Ag antiviral nanomaterial coating in the facemasks should minimize the risk of transmission of infectious agents.

## 5. Conclusions

**Figure 7:**
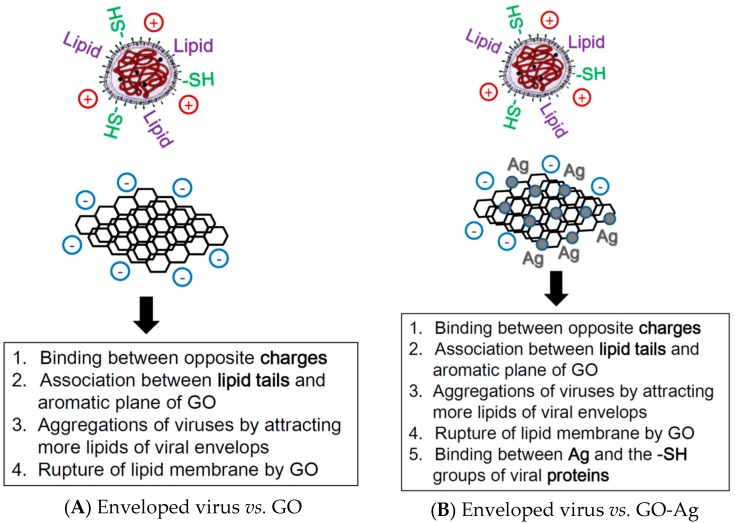

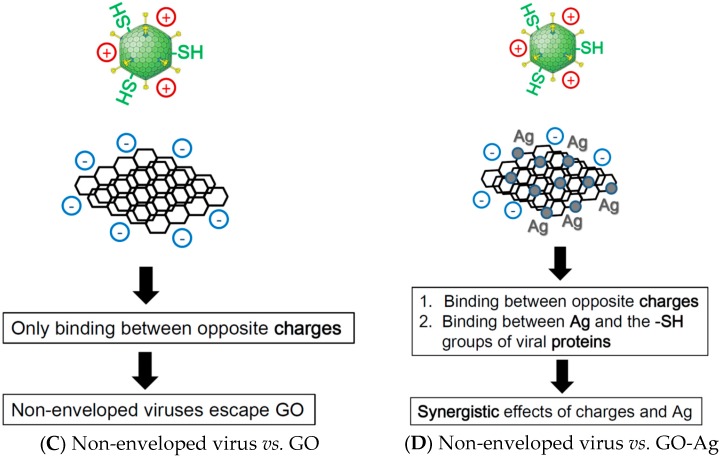
Schematic for the antiviral mechanisms of (**A**) graphene oxide (GO) against the enveloped virus; (**B**) graphene-silver nanocomposites (GO-Ag) against the enveloped virus; (**C**) GO against the non-enveloped virus; (**D**) GO-Ag against the non-enveloped virus.

GO sheets with silver particles exhibited antiviral activity against both enveloped viruses and non-enveloped viruses, whereas GO sheets alone could only inhibit the infection of enveloped viruses at non-cytotoxic concentrations. Although the antiviral mechanism requires further research, the unique structure of graphene oxide sheets could contribute to the inhibition of infection by feline coronavirus with a lipid envelope. To inhibit the infection of infectious bursal disease virus without a lipid envelope, the conjugation between the sulfur groups of viral proteins and the silver particles on the surface of graphene oxide could be important. Studies on the interactions between GO and lipid membranes showed that negatively charged GO can absorb to positively charged lipid membranes and induce the rupture of lipid membranes [28]. The lipid tails exposed from the ruptured lipid membrane would associate strongly to the aromatic plane of the GO sheet [29]. The interactions between GO and lipid membrane can attract the absorption of more lipid membranes [28]. Therefore, a tentative model was proposed for the antiviral mechanisms of GO and GO-Ag against enveloped and non-enveloped viruses (Figure 7).

## Figures and Tables

**Table 1:** Virus inhibition assay of the viruses incubated with GO or GO-Ag.

		mg/mL *	TCID_50_/mL	Inhibition %
Exp 1	FCoV		10^6^	
	FCoV + GO	100	10^5^	17
		10	4.7 × 10^5^	5.5
		1	3.7 × 10^5^	7.2
	FCoV + GO-Ag	100	10^5^	17
		10	1.6 × 10^5^	13
		1	3.2 × 10^5^	8.4
Exp 2	FCoV		4.7 × 10^4^	
	FCoV + GO	0.1	8.1 × 10^3^	16.3
	FCoV + GO-Ag	0.1	3.7 × 10^3^	**24.8**
Exp 3	IBDV		9 × 10^5^	
	IBDV + GO	1	9.5 × 10^5^	−0.4
	IBDV + GO-Ag	1	4 × 10^4^	**22.7**

* Concentration of GO or GO-Ag in mg/mL. Inhibition % = (log_10_ (TCID_50_/mL of virus) − log_10_ (TCID_50_/mL of treatment))/log_10_ (TCID_50_/mL of virus) × 100%; FCoV: feline coronavirus; GO: graphene oxide; GO-Ag: graphene oxide with Ag particles; IBDV: infectious bursal disease virus.

**Table 2:** Infectivity of viruses inoculated with graphene oxide with silver.

	Concentration (mg/mL)				
	1	0.5	0.25	0.125	0.0625
**IBDV: 9 × 10^3^ TCID_50_/mL**					
IBDV + GO	100%	100%	100%	100%	N.A.
IBDV + GO-Ag	75%	87.5%	100%	100%	N.A.
**IBDV: 9 × 10^2^ TCID_50_/mL**					
IBDV + GO	100%	100%	100%	100%	100%
IBDV + GO-Ag	0%	0%	0%	0%	37.5%

Infectivity % = (number of wells with virus-infected cells/number of wells with virus-inoculated cells) × 100%; GO: graphene oxide; GO-Ag: graphene oxide with Ag particles; IBDV: infectious bursal disease virus; N.A.: no available.

## References

[1] Albrecht M.A., Evan C.W., Raston C.L. (2006). Green chemistry and the health implications of nanoparticles. Green Chem..

[2] Kim J.S., Kuk E., Yu K.N., Kim J.H., Park S.J., Lee H.J. (2007). Antimicrobial effects of silver nanoparticles. Nanomed. Nanotechnol. Biol. Med..

[3] Gong P., Li H., He X., Wang K., Hu J., Tan W. (2007). Preparation and antibacterial activity of Fe_3_O_4_@Ag nanoparticles. Nanotechnology.

[4] Elechiguerra J.L., Burt J.L., Morones J.R., Camacho-Bragado A., Gao X., Lara H.H., Yacaman M.J. (2005). Interaction of silver nanoparticles with HIV-1. J. Nanobiotechnol..

[5] Lara H.H., Ayala-Nunez N., Ixtepan-Turrent L., Rodriguez-Padilla C. (2010). Mode of antiviral action of silver nanoparticles against HIV-1. J. Nanobiotechnol..

[6] Lara H.H., Ixtepan-Turrent L., Garza-Trevino E.N., Rodriguez-Padilla C. (2010). PVP-coated silver nanoparticles block the transmission of cell-free and cell-associated HIV-1 in human cervical culture. J. Nanobiotechnol..

[7] Lu L., Sun R.W., Chen R., Hui C.K., Ho C.M., Luk J.M., Lau G.K., Che C.M. (2008). Silver nanoparticles inhibit hepatitis B virus replication. Antivir. Ther..

[8] Baram-Pinto D., Shukla S., Perkas N., Gedanken A., Sarid R. (2009). Inhibition of herpes simplex virus type 1 infection by silver nanoparticles capped with mercaptoethane sulfonate. Bioconj. Chem..

[9] Sun L., Singh A.K., Vig K., Pillai S.R., Singh S.R. (2008). Silver nanoparticles inhibit replication of respiratory syncytial virus. J. Biomed. Nanotechnol..

[10] Rogers J.V., Parkinson C.V., Choi Y.W., Speshock J.L., Hussain S.M. (2008). A preliminary assessment of silver nanoparticle inhibition of monkeypox virus plaque formation. Nanoscale Res. Lett..

[11] Speshock J.L., Murdock R.C., Braydich-Stolle L.K., Schrand A.M., Hussain S.M. (2010). Interaction of silver nanoparticles with Tacaribe virus. J. Nanobiotechnol..

[12] Mehrbod P., Motamed N., Tabatabaian M., Soleimani E.R., Amini E., Shahidi M., Kheiri M.T. (2009). *In vitro* antiviral effect of “Nanosilver” on influenza virus. DARU.

[13] Mori Y., Ono T., Miyahira Y., Nguyen V.Q., Matsui T., Ishihara M. (2013). Antiviral activity of silver nanoparticles/chitosan composites against H1N1 influenza A virus. Nanoscale Res. Lett..

[14] Xiang D., Chen Q., Pang L., Zheng C.-L. (2011). Inhibitory effects of silver nanoparticles on H1N1 influenza A virus *in vitro*. J. Virol. Methods.

[15] Rao C.N.R., Sood A.K., Voggu R., Subrahmanyam K.S. (2010). Some novel attributes of graphene. J. Phys. Chem. Lett..

[16] Faria A.F., Martinez D.S.T., Meira S.M.M., Moraes A.C.M., Brandelli A., Filho A.G.S., Alves O.L. (2014). Anti-adhesion and antibacterial activity of silver nanoparticles supported on graphene oxide sheets. Colloid Surf. B Biointerfaces.

[17] Gurunathan S., Han J.W., Dayem A.A., Eppakayala V., Kim J.-H. (2012). Oxidative stress-mediated antibacterial activity of graphene oxide and reduced graphene oxide in *Pseudomonas aeruginosa*. Int. J. Nanomed..

[18] Liu S., Hu M., Zeng T.H., Wu R., Jiang R., Wei J., Wang L., Kong J., Chen Y. (2012). Lateral dimension-dependent antibacterial activity of graphene oxide sheets. Langmuir.

[19] Sametband M., Kalt I., Gedanken A., Sarid R. (2014). Herpes simplex virus type-1 attachment inhibition by functionalized graphene oxide. ACS Appl. Mater. Interfaces.

[20] Ye S., Shao K., Li Z., Guo N., Zuo Y., Li Q., Lu Z., Chen L., He Q., Han H. (2015). Antiviral activity of graphene oxide: How sharp edged structure and charge matter. ACS Appl. Mater. Interfaces.

[21] Pedersen N.C., Black J.W., Boyle J.F., Evermann J.F., McKeirnan A.J., Ott R.L. (1984). Pathogenic differences between various feline coronavirus isolates. Adv. Exp. Med. Biol..

[22] Rekha K., Sivasubramanian C., Chung I.-M., Thiruvengadam M. (2014). Growth and replication of infectious bursal disease virus in the DF-1 cell line and chicken embryo fibroblasts. Biomed. Res. Int..

[23] Hummers W.S., Offeman R.E. (1958). Preparation of graphitic oxide. J. Am. Chem. Soc..

[24] Reed L.J., Muench H. (1938). A simple method of estimating fifty percent endpoints. Am. J. Epidemiol..

[25] R Core Team (2013). R: A Language and Environment for Statistical Computing.

[26] Tang J., Chen Q., Xu L., Zhang S., Feng L., Cheng L., Xu H., Liu Z., Peng R. (2013). Graphene oxide-silver nanocomposites as a highly effective antibacterial agent with species-specific mechanisms. ACS Appl. Mater. Interfaces.

[27] Li Y., Leung P., Yao L., Song Q.W., Newton E. (2006). Antimicrobial effect of surgical masks coated with nanoparticles. J. Hosp. Infect..

[28] Frost R., Jonsson G.E., Chakarov D., Svedhem S., Kasemo B. (2012). Graphene oxide and lipid membranes: Interactions and nanocomposite structures. Nano Lett..

[29] Rui L., Liu J., Li J., Weng Y., Dou Y., Yuan B., Yang K., Ma Y. (2015). Reduced graphene oxide directed self-assembly of phospholipid monolayers in liquid and gel phases. Biochim. Biophys. Acta.

